# A G-CHAM program and policy development curriculum using simulated executive review in undergraduate public health nursing education

**DOI:** 10.3389/fpubh.2026.1909265

**Published:** 2026-10-09

**Authors:** Ryo Horiike, Izumi Kondo, Harumi Bando

**Affiliations:** Department of Public Health Nursing, School of Medicine, Nara Medical University, Nara, Japan

**Keywords:** community health assessment, competency, G-CHAM, GIS, program and policy development, public health nursing, simulation based education

## Abstract

**Background:**

Public health nurses in Japan commonly work in administrative settings and are expected to translate community health assessment findings into feasible programs and policies through organizational decision making, negotiation, and budgeting. However, undergraduate public health nursing education provides limited opportunities for students to experience this administrative program and policy development process. This study describes a G-CHAM program and policy development curriculum using simulated executive review and presents an initial assessment of changes in students’ self assessed competency.

**Methods:**

The curriculum was implemented in a fourth year undergraduate public health nursing course. Before this section, students had completed G-CHAM activities including QGIS based community assessment, digitally supported windshield survey fieldwork, and identification of community health issues. The program and policy development section included introductory instruction, preparation of a program planning document and one page proposal visual, and a 90 min simulated executive review session incorporating structured debriefing. Four administrative staff members with public sector experience participated as an executive review panel. Outcomes were assessed immediately before and after the section using seven self assessment items based on the 2024 revised Model Core Curriculum for Public Health Nursing Education in Japan.

**Results:**

Nineteen students participated, and 18 provided linkable pre and post responses. The median total self assessed competency score increased from 9.50 to 26.50. All seven item scores increased significantly after Holm correction. Before the section, no students reached level 4 or higher on any item; after the section, the proportions reaching level 4 or higher ranged from 50.0 to 83.3%.

**Conclusion:**

Students reported higher self assessed competency after completing the G-CHAM program and policy development section. The findings should be interpreted as short term perceived learning from a single group educational evaluation rather than evidence of objective competency improvement or a causal effect of the simulated executive review alone. Future studies should incorporate objective performance measures, comparison groups, and longer term follow up.

## Introduction

Public health nurses are expected to assess population level health needs, identify community health issues, and translate assessment findings into feasible programs and policies. In public health nursing education, community health assessment is therefore not only a technical exercise in data collection but also a foundational process for developing practical judgment about where, for whom, and how public health interventions should be implemented. Community health assessment models and tools have been used to structure community level data collection and interpretation in nursing practice and education (1, 2). Previous Japanese studies have also suggested that structured community diagnosis exercises can improve students’ understanding of community characteristics, although students may continue to experience difficulty in integrating diverse data sources and linking assessment findings to practical planning (3, 4).

In Japan, this educational issue is closely linked to the employment context of public health nurses. According to national health administration statistics, 44,825 of 63,536 employed public health nurses worked in public administrative settings, including public health centers, prefectural governments, and municipalities, accounting for approximately 70.6% of all employed public health nurses (5). The Public Health Nurses, Midwives and Nurses Act legally protects the title of public health nurse, but public health nurses working in local governments must also function as administrative staff members within organizational decision making systems (6). Previous research has indicated that public health nurses in administrative organizations require not only professional health expertise but also administrative perspectives, skills, and understanding of governmental systems (7). Undergraduate public health nursing education should therefore provide opportunities for students to learn how community assessment findings are translated into administratively feasible programs and policies.

Geographic information systems have been introduced as educational tools to support community health assessment because they allow students to visualize population distribution, health resources, environmental conditions, and spatial inequities. Earlier work in public health nursing education reported that GIS can help nursing students develop knowledge and skills related to population health assessment and intervention planning (8). In Japan, GIS based community diagnosis curricula for public health nursing students have been implemented, and students were reported to learn technical GIS operations as well as public health nursing perspectives such as identifying community strengths and health issues (9). However, GIS based education may remain limited if spatial assessment is not explicitly connected to administrative decision making, program planning, and policy development.

Simulation based education has increasingly been used in community and public health nursing education. Prior studies have applied simulation to public health nursing skills, health guidance, virtual community health nursing, and public health role performance (10–13). A recent scoping review reported that simulation in community and public health nursing education has addressed diverse topics and learning outcomes, while also noting variability in simulation modalities, evaluation methods, and outcome measures (14). These studies suggest that simulation can provide structured opportunities for students to apply knowledge in realistic scenarios, although the evidence base remains varied.

However, this literature has generally focused on specific public health nursing activities or professional roles rather than the administrative process of translating GIS based community assessment into program proposals, budget related justification, and organizational review. Thus, the educational gap addressed here is the limited description of curricula that explicitly connect GIS based community assessment, program and policy development, and simulated administrative review within a single learning sequence.

G-CHAM, the GIS based Community Health Assessment Method, was developed to support public health nurses and students in conducting community health assessment using GIS and linking assessment findings to public health nursing practice (15). In the present curriculum, students used G-CHAM to assess community characteristics, identify health issues, prepare program planning documents, create one page proposal visuals, and participate in simulated administrative review. The distinctive feature was the integration of these steps into a sequential program and policy development process involving administrative staff with practical experience in planning, budgeting, organizational management, and administrative review. This study aimed to describe the design and implementation of the G-CHAM program and policy development section and to assess changes in students’ self assessed competency before and after the educational activity.

### Pedagogical framework and learning objectives

The educational design of the G-CHAM program and policy development section was informed by three complementary perspectives: competency based education, simulation based education, and community health assessment and planning consistent with the Community as Partner Model. Competency based education emphasizes alignment among learning outcomes, educational activities, and assessment with the competencies learners are expected to acquire (16). This perspective was appropriate because students were expected not only to acquire knowledge but also to use community assessment findings, explain the rationale for a proposed program, respond to administrative questions, and understand how public health nursing practice is translated into feasible administrative action.

The community health assessment component was grounded in G-CHAM and was consistent with the Community as Partner Model. The Community as Partner Model provides a structured way of viewing communities as systems composed of interrelated subsystems and has been used in population based community health nursing education to guide assessment data collection and project development (1, 2). G-CHAM operationalized this orientation by using GIS to help students examine community characteristics, identify health issues, and consider program responses based on spatially and socially situated evidence (8, 9, 15). In this curriculum, community assessment was positioned not as an endpoint but as the basis for program and policy development.

Simulation based education was incorporated to allow students to experience a situation approximating an administrative decision making process. The Healthcare Simulation Standards of Best Practice describe simulation as an educational strategy that can include preparation, prebriefing, a simulation experience, and debriefing to support structured learning (17). In this activity, students assumed the role of administrative public health nurses proposing a program, while the executive review panel assumed senior administrative roles. This situated structure was intended to help students engage with practical questions about feasibility, budget, organizational accountability, stakeholder explanation, and implementation.

Debriefing was included after the simulated executive review. Prior reviews of simulation debriefing in nursing education have emphasized that debriefing allows learners to reflect on performance, connect experience with underlying principles, and identify implications for future practice (18). In this curriculum, debriefing focused on the clarity of the proposal, the logic of the explanation, the consistency between the identified community health issue and the proposed program, and the administrative viewpoints raised by the executive review panel.

The learning objectives were aligned with the 2024 revised Model Core Curriculum for Public Health Nursing Education in Japan. Specifically, the assessment items corresponded to the third layer 5–1-4, which concerns program development for solving community health issues and promoting health. The seven learning objectives were to enable students to develop a program, develop an evaluation plan, explain consultation and consensus building, understand program planning based on laws and budgets, understand implementation and progress management, explain the significance of disseminating outcomes, and explain the role of public health nurses in program and policy development (19). These objectives were used to structure the educational activity and evaluate students’ self assessed competency levels.

### Curriculum design and learning environment

This educational activity was implemented in the course “Public Health Nursing Activity Development Theory” at the Department of Nursing, School of Medicine, Nara Medical University, in the 2026 academic year. The elective course enrolled 19 fourth year nursing students in the public health nurse track. All were undergraduate students without prior professional employment experience and had not yet begun their public health nursing practicum. They had no prior experience in administrative practice, public health nursing practice, program or budget planning, or simulation based education. According to the syllabus, the course aimed to enable students to learn the sequential process of administrative public health nursing practice by using G-CHAM to identify health issues from community characteristics and develop program plans through program planning and simulated executive review.

The course consisted of 30 sessions, each lasting 90 min. The first half focused on the purpose and methods of community health assessment, QGIS operation, and assessment of community characteristics, including administration and politics, population statistics, history, ethnicity and values, the physical environment, health care and social welfare, economy, transportation and safety, education and recreation, communication and information, and additional GIS based data collection. Subsequent sessions included G-CHAM DX fieldwork, a digitally supported windshield survey using geolocation and QGIS, followed by integration of field observations with other community assessment data and identification of community health issues. Thus, before the program and policy development section, students had completed QGIS based community assessment, fieldwork, and health issue identification.

The program and policy development component was implemented from Session 26 onward as the final stage of the G-CHAM learning process. The pre assessment was administered immediately before Session 26. Session 26 introduced the relationships among policy, policy measures, programs, budgeting, and administrative decision making and initiated preparation of the program planning document. Sessions 27–29 were devoted primarily to student led group work on the program planning document and one page proposal visual, with faculty guidance provided only when requested or when clarification was needed. Students worked in five groups of three to four and developed proposals based on health issues identified through the preceding G-CHAM activities. The program planning document served as a detailed internal reference for each team, whereas the one page proposal visual served as the primary communication material for the executive review.

On May 22, 2026, four consecutive class sessions were conducted. Sessions 27–29 were used for program planning and proposal visual preparation, and Session 30 was used for the 90 min Special Version of the simulated executive review. The post assessment was administered immediately after all Session 30 activities. Accordingly, the pre post assessment covered the entire program and policy development section rather than the simulated executive review alone.

### Special version of the simulated executive review

The distinctive feature of this educational activity was the Special Version of the G-CHAM simulated executive review. In the standard format, students alternate between a public health nurse team proposing a program and an executive review team examining it from administrative perspectives. In the Special Version, four administrative staff members with public sector experience participated as an executive review panel, while students also experienced the executive reviewer role through role rotation.

The panel consisted of four administrative staff members from the Educational Affairs Division of Nara Medical University, assuming the roles of department director, medical deputy director, administrative deputy director, and finance officer. All had professional experience in prefectural government or a public university corporation; experience in general and policy planning, finance and budgeting, and organizational management; and experience both undergoing and conducting program or budget review. Roles were assigned according to each member’s prior administrative experience. Although they were not current municipal public health executives, they represented administrative perspectives relevant to feasibility, accountability, coordination, and budgeting.

Before the simulation, the panel members participated in a one hour orientation based on the G-CHAM Special Version protocol. The orientation covered the educational purpose, preceding G-CHAM activities, assigned roles and review perspectives, questioning rules, timing, session flow, and debriefing procedure. Panel members were instructed that the purpose was to promote learning through administrative questioning rather than to grade the students. They first viewed each group’s one page proposal visual during the simulation.

Five student groups participated, and all five were directly reviewed at least once by the four administrative panel members. Because there were five groups, two groups also participated in peer executive review during role rotation. Across the session, all students experienced both the public health nurse and executive reviewer perspectives. During each review, the public health nurse team explained the necessity, target population, rationale, implementation plan, expected outcomes, evaluation approach, and budgetary considerations of its proposed program. The team presented a one page proposal visual; the detailed program planning document remained with the team as a reference for responding to questions.

The review process was standardized in terms of role assignments, review perspectives, presentation materials, timing, questioning rules, and session flow. Panelists were not given scripted questions; they formulated questions according to their assigned roles and each proposal. Because community health issues and proposed programs differed among groups, question content and difficulty were not standardized. Questions addressed policy consistency, public health relevance, organizational feasibility, interdepartmental coordination, accountability, and budget justification.

Preparatory instructions specified in the G-CHAM Special Version protocol were provided to students in advance and were not included in the 90 min Session 30. The planned activity time within Session 30 was 78 min: one 34 min representative round (1 min proposal display, 5 min reviewer preparation, 13 min review, and 15 min debriefing) followed by two 22 min role rotation rounds (1 min proposal display, 5 min reviewer preparation, 13 min review, and 3 min debriefing each). The remaining time was used for movement between tables and reconfiguration between rounds. The first author facilitated the debriefing. All students could observe the representative debriefing. Using a whiteboard, the public health nurse and executive review teams reflected on predefined learning goals, recording strengths and areas for improvement; the administrative panel also explained the rationale for its questions and provided feedback on administrative review perspectives and student responses. This shared debriefing was intended to allow subsequent groups to apply the feedback during later rounds. Detailed implementation materials, including the program planning template, proposal visual guidance, Special Version procedure, reviewer roles and perspectives, questioning rules, timing, and debriefing procedure, are publicly available without login at https://sites.google.com/view/g-cham/wahtisg-cham/policymaking.

### Assessment and outcome measures

Student learning outcomes were assessed using a pre post self assessment questionnaire based on the 2024 revised Model Core Curriculum for Public Health Nursing Education in Japan, specifically third layer 5-1-4 on program development for solving community health issues and promoting health (19). The seven competency items were taken directly from this curriculum domain without modification.

The seven items assessed whether students could develop a program, develop an evaluation plan, explain the significance of consultation and consensus building, understand program planning based on laws and budgets, understand implementation and progress management, explain the significance of disseminating program outcomes, and explain the role of public health nurses in program and policy development. They were used as curriculum aligned indicators of self assessed competency rather than direct measures of observed performance. No separate psychometric validation was conducted for this educational evaluation.

The Model Core Curriculum defines four attainment levels: having the necessary knowledge; being able to integrate knowledge and consider the issue; being able to perform simulated practice based on evidence; and being able to perform practice based on evidence. For this educational evaluation, an additional category, “no knowledge,” was added below the lowest curriculum level, resulting in a five point ordinal scale: 1, no knowledge; 2, has the necessary knowledge; 3, can integrate knowledge and think about the issue; 4, can perform simulated practice based on evidence; and 5, can perform practice based on evidence. Level 4 or higher was defined for the present educational evaluation as attainment of at least the simulated practice level because it marks the transition from knowledge and cognitive integration to simulated performance; this threshold is not specified by the Model Core Curriculum.

The primary outcome was the seven item total score (7–35), used as an exploratory summary index of related program and policy development competencies rather than a validated psychometric scale score. Secondary outcomes were each item score, the number of items for which each student reached level 4 or higher, and the proportion of students reaching level 4 or higher for each item.

### Data collection and ethical considerations

Data were collected as part of routine educational evaluation. The pre assessment was administered immediately before Session 26, after the preceding G-CHAM community assessment activities but before the program and policy development section began. The post assessment was administered immediately after completion of Session 30. Both assessments used Google Forms.

Pre and post responses were linked using a self generated keyword without names, student numbers, or other directly identifiable information. For the post assessment, students responded individually on their own smartphones after class, and the instructor could not view individual input. The linking keywords could not be used by the instructor to identify students. Students were informed that participation was voluntary, that individual respondents could not be identified, and that responses would not affect grades or academic standing. No demographic or sensitive personal information was collected. Eighteen of 19 students provided linkable pre and post responses; one student was excluded because a linkable pair was not available.

Participation in the questionnaire was explained as part of course improvement and was not used for grading. Students were informed that questionnaire responses would not affect course evaluation or academic standing. This activity was implemented as a regular educational activity and evaluated for educational improvement purposes. In Japan, the Ethical Guidelines for Medical and Biological Research Involving Human Subjects apply to medical and biological research involving human subjects, and the current national guidelines were implemented in 2021 by integrating previous guidelines for medical and health research and human genome or gene analysis research (20). The present activity did not collect clinical information, biological samples, identifiable personal information, or data beyond the scope of routine course evaluation. Therefore, it was treated as outside the scope of ethics committee review under the relevant Japanese research ethics framework.

### Statistical analysis

Statistical analyses were conducted using jamovi version 2.6 and R version 4.4 (21, 22). Descriptive statistics were calculated for each item and the total score. Medians and interquartile ranges were the primary summaries for ordinal item scores; means and standard deviations were also reported. The total score was used as an exploratory summary index because all seven items were drawn from the same program and policy development domain and was not interpreted as a validated psychometric scale score.

Cronbach’s alpha was calculated descriptively to examine internal consistency within this sample and was not interpreted as evidence of construct validity, criterion validity, or responsiveness (23). Pre and post scores were compared using the Wilcoxon signed rank test, with Holm adjustment for item level comparisons. Rank biserial correlation was calculated as an effect size; because the comparison direction was pre minus post, negative values indicated higher post scores.

The number of items reaching level 4 or higher was compared before and after the educational activity using the Wilcoxon signed rank test. For each item, changes in the proportion of students reaching level 4 or higher were examined using an exact McNemar approach based on the binomial test for discordant pairs. Holm correction was applied to these item level comparisons. All statistical tests were two sided, and the significance level was set at 0.05.

## Results and assessment

Among the 19 students, 18 provided linkable pre and post responses and were included in the analysis; no missing values were observed. Descriptive statistics are shown in Table 1. Cronbach’s alpha was 0.94 at both time points and is reported descriptively as internal consistency within this sample.

**Table 1:** Descriptive statistics of competency scores before and after the G-CHAM program and policy development.

Item	Competency item	Pre median [IQR]	Post median [IQR]	Pre mean ± SD	Post mean ± SD
Q1	Develop a program to solve community health issues and promote health	2.00 [1.00–2.00]	4.00 [3.00–4.00]	1.89 ± 0.76	3.83 ± 0.86
Q2	Develop an evaluation plan for the program	1.00 [1.00–2.00]	4.00 [3.25–4.75]	1.50 ± 0.62	3.94 ± 0.87
Q3	Explain the significance of consultation, negotiation, and consensus building with relevant organizations, departments, and community members	2.00 [1.00–2.00]	4.00 [3.00–4.00]	1.72 ± 0.75	3.67 ± 0.77
Q4	Understand program planning based on laws, ordinances, local basic policies, plans, and budgets	1.00 [1.00–2.00]	3.50 [3.00–4.00]	1.44 ± 0.70	3.56 ± 1.10
Q5	Understand implementation methods and progress management for the planned program	1.00 [1.00–1.75]	4.00 [3.00–4.00]	1.39 ± 0.70	3.67 ± 1.03
Q6	Explain the significance of disseminating program outcomes to relevant organizations, departments, and community members	1.00 [1.00–2.00]	4.00 [3.25–4.00]	1.56 ± 0.78	3.89 ± 0.83
Q7	Explain the role of public health nurses in program and policy development	2.00 [1.00–2.00]	4.00 [4.00–5.00]	1.72 ± 0.75	4.17 ± 0.86
Total score	Sum of the seven competency items	9.50 [8.00–14.00]	26.50 [24.00–29.75]	11.22 ± 4.32	26.72 ± 5.41

The total self assessed competency score increased from a median of 9.50 before the section to 26.50 after the section; the mean increased from 11.22 to 26.72. Individual changes are shown in Figure 1. The Wilcoxon signed rank test was statistically significant (*p* = 0.000195), and the rank biserial correlation was −1.00.

**Figure 1:**
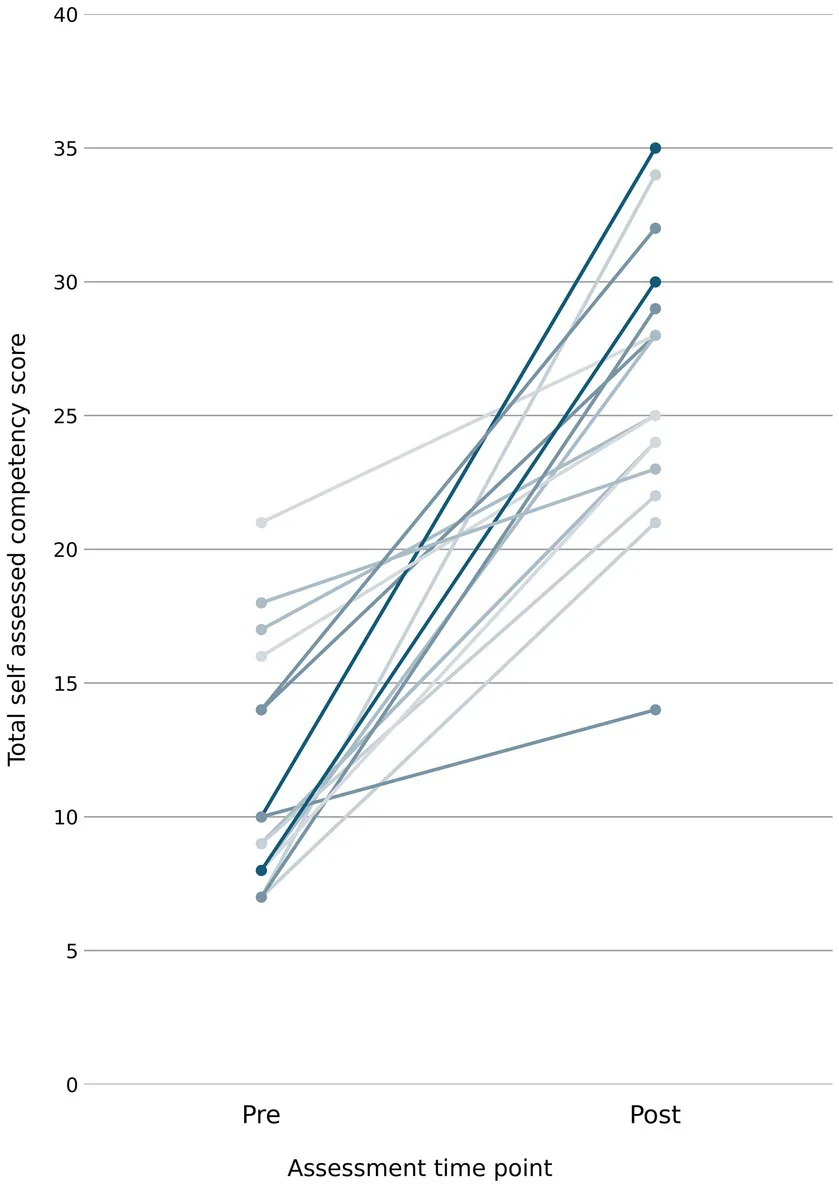
Individual changes in the seven item total self assessed competency score before and after the G-CHAM program and policy development section. Each line represents one of the 18 students with linkable pre and post responses. The possible total score ranged from 7 to 35.

Item level analyses showed higher post scores across all seven items (Table 2). Sixteen to 18 students improved on each item, 0 to 2 were unchanged, and none decreased. Wilcoxon *p* values ranged from 0.000175 to 0.000397, and all remained significant after Holm correction. The rank biserial correlation reached the boundary value of −1.00 for each comparison because all non tied pairs changed toward higher post scores; these estimates should be interpreted cautiously given the small sample.

**Table 2:** Pre post comparison of self assessed competency scores using the Wilcoxon signed rank test.

Item	Pre median [IQR]	Post median [IQR]	Improved, *n*	Unchanged, *n*	Decreased, *n*	Wilcoxon *p*	Holm adjusted *p*	Rank biserial correlation
Q1	2.00 [1.00–2.00]	4.00 [3.00–4.00]	17	1	0	0.000252	0.001513	−1.00
Q2	1.00 [1.00–2.00]	4.00 [3.25–4.75]	17	1	0	0.000252	0.001513	−1.00
Q3	2.00 [1.00–2.00]	4.00 [3.00–4.00]	16	2	0	0.000378	0.001513	−1.00
Q4	1.00 [1.00–2.00]	3.50 [3.00–4.00]	16	2	0	0.000397	0.001513	−1.00
Q5	1.00 [1.00–1.75]	4.00 [3.00–4.00]	17	1	0	0.000257	0.001513	−1.00
Q6	1.00 [1.00–2.00]	4.00 [3.25–4.00]	16	2	0	0.000382	0.001513	−1.00
Q7	2.00 [1.00–2.00]	4.00 [4.00–5.00]	18	0	0	0.000175	0.001225	−1.00

No confidence intervals were returned by jamovi for the rank biserial correlations, which were boundary estimates of −1.00.

The number of items reaching level 4 or higher increased from a mean of 0.00 before the section to 4.67 after the section, with a post median of 5.00 (Wilcoxon signed rank *p* = 0.000352). Item level attainment is summarized in Table 3 and Figure 2.

**Table 3:** Proportion of students reaching self assessed competency level 4 or higher before and after the G-CHAM program and policy development section.

Item	Pre reached level 4 or higher, *n* (%)	Post reached level 4 or higher, *n* (%)	Gained, *n*	Lost, *n*	Exact McNemar *p*	Holm adjusted *p*
Q1	0 (0.0)	12 (66.7)	12	0	0.000488	0.001953
Q2	0 (0.0)	13 (72.2)	13	0	0.000244	0.001465
Q3	0 (0.0)	11 (61.1)	11	0	0.000977	0.002930
Q4	0 (0.0)	9 (50.0)	9	0	0.003906	0.003906
Q5	0 (0.0)	11 (61.1)	11	0	0.000977	0.002930
Q6	0 (0.0)	13 (72.2)	13	0	0.000244	0.001465
Q7	0 (0.0)	15 (83.3)	15	0	0.000061	0.000427

**Figure 2:**
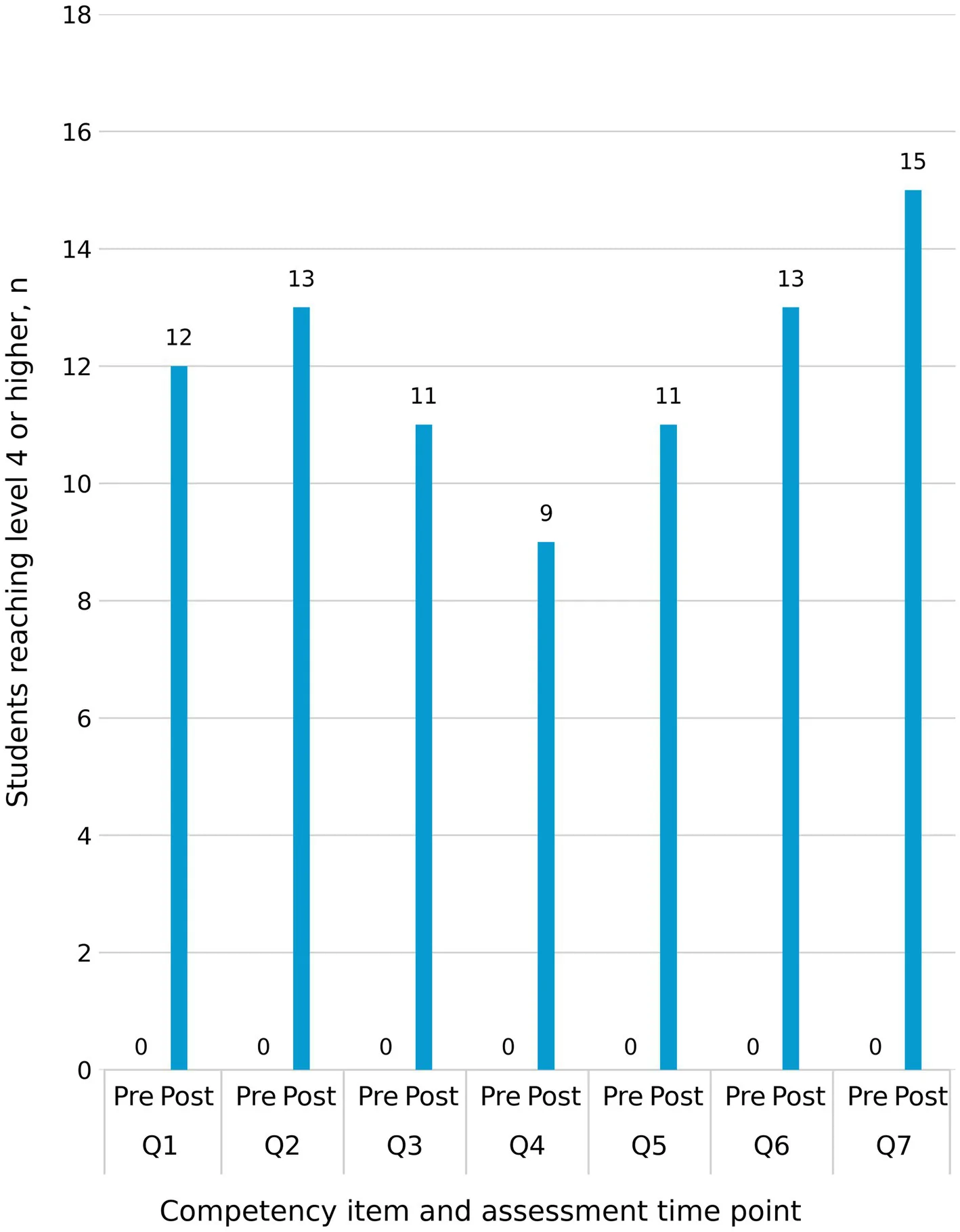
Number of students reaching self assessed competency level 4 or higher for each of the seven competency items before and after the G-CHAM program and policy development section (*n* = 18). Level 4 represents the ability to perform simulated practice based on evidence.

Before the section, no student reached level 4 or higher on any item. After the section, the proportion reaching level 4 or higher ranged from 50.0 to 83.3%. The highest proportion was for explaining the role of public health nurses in program and policy development, and the lowest was for planning based on laws, policies, plans, and budgets. Exact McNemar analyses remained significant for all seven items after Holm correction.

Overall, post section scores indicated higher self assessed competency across the seven curriculum aligned items. The comparatively lower attainment for the item concerning laws, policies, plans, and budgets identifies an area that may require additional instructional reinforcement.

## Discussion

This study described a G-CHAM program and policy development section integrating GIS based community health assessment, program planning, proposal visual preparation, simulated executive review, and debriefing. Students reported higher self assessed competency across all seven curriculum aligned items after completing the section. Because the pre post assessment covered the entire program and policy development section, these changes should not be interpreted as effects of the simulated executive review alone.

The findings are consistent with previous work suggesting that GIS can support population level thinking and community health assessment in public health nursing education (8, 9). The present activity extended this direction by positioning GIS based community assessment as the starting point for program and policy development. G-CHAM was therefore used not only as a mapping or data visualization tool but also as part of a sequential learning process from community assessment to health issue identification, program planning, proposal communication, and administrative review (15).

The results also align with the broader literature on simulation based education in community and public health nursing. Prior studies have used simulation to support public health nursing skills, health guidance, virtual community health nursing learning, and public health role performance (10–13). A recent scoping review reported that simulation in community and public health nursing education has been applied to diverse topics and learning outcomes, while also noting variability in design and evaluation methods (14). The present activity contributes to this area by focusing on the administrative review process through which public health nurses must explain, justify, and revise proposed programs within organizational and financial constraints.

A distinctive feature was the participation of administrative staff as the executive review panel. Their experience in public sector planning, budgeting, organizational management, and administrative review may have increased the administrative authenticity of the simulation. Although they were not current municipal public health executives, their experience on both sides of program and budget review provided perspectives relevant to feasibility, accountability, coordination, and resource allocation. The panel’s questions emphasized feasibility, accountability, alignment with plans and policies, and the use of public resources, illustrating that program and policy development requires not only identifying community health issues but also justifying priority, funding, implementation, and evaluation.

The relatively high post section attainment for the item related to explaining the role of public health nurses may reflect increased perceived understanding of this professional role within administrative systems. This is educationally relevant because community assessment education can be strengthened when it is connected to planning, implementation, and evaluation rather than ending with data interpretation alone (1, 2). By assuming the role of administrative public health nurses and responding to questions from an executive review panel, students could experience the gap between identifying a health issue and obtaining organizational support for a proposed response.

This point is particularly relevant in Japan. National statistics show that approximately 70.6% of employed public health nurses work in public administrative settings, including public health centers, prefectural governments, and municipalities (5). Although the Public Health Nurses, Midwives and Nurses Act legally protects the professional title of public health nurse, public health nurses in local governments are also required to work as members of administrative organizations (6). Hirano et al. reported that public health nurses in administrative organizations require the perspective and skills of administrative staff members, as well as an understanding of governmental functions and systems (7). The present activity may therefore have educational value as an early opportunity for students to experience administrative reasoning, negotiation, and decision making processes they may encounter after employment.

The specific executive review structure reflects the Japanese administrative context, but the core educational sequence may be adaptable to other public health systems. Community assessment, program proposal development, presentation to decision makers, administrative questioning, and structured debriefing could be implemented with locally relevant public health managers, administrators, finance personnel, or intersectoral partners. Transferability therefore depends on adapting the reviewer roles while preserving the sequence of evidence informed proposal development and organizational review.

The item with the lowest post activity attainment was understanding program planning based on laws, ordinances, local policies, plans, and budgets. This result suggests that administrative and financial reasoning may require more explicit instruction and repeated practice. Although students prepared program proposals and responded to questions from the finance officer and other executive roles, the lower attainment after this initial section indicates that legal, policy, and budgetary conditions may require continued learning beyond a single educational sequence. This interpretation is consistent with previous research suggesting that policy development, health service development, and administrative competency among public health nurses require structured competency assessment, continuing professional development, and workplace based learning after graduation (24, 25).

Using the 2024 revised Model Core Curriculum aligned the assessment with nationally defined competency expectations (19). However, the outcomes represent self assessment rather than observed performance. Higher post section scores therefore indicate increased perceived competency and should not be interpreted as direct evidence of objective competency improvement. Students’ understanding of the scope of the competencies may also have changed through the educational activities, potentially influencing post ratings.

Overall, this educational activity provides a structured approach for connecting GIS based community health assessment with administrative program and policy development. G-CHAM supported a sequence from community assessment and health issue identification to program development, proposal communication, administrative review, and debriefing (15). Further studies using larger and multicenter samples, comparison groups, objective performance assessments, and longer follow up are needed to examine educational outcomes and transferability.

### Constraints and limitations

This study has several constraints and limitations. First, the single group pre post design without a comparison group does not establish a causal effect of the G-CHAM program and policy development section or of the simulated executive review specifically. The preceding G-CHAM community assessment, fieldwork, and health issue identification had been completed before the pre assessment and formed the prerequisite learning context. The pre post comparison covered the subsequent sequence of introductory instruction, program planning, proposal preparation, simulated executive review, and debriefing; the contribution of each component cannot be separated.

Second, the analysis included 18 students from one public health nursing course at a single university. The findings should therefore be interpreted as an initial educational evaluation rather than evidence of generalizable effectiveness. Replication with larger and multicenter samples is needed.

Third, outcomes were based on curriculum aligned self assessment items rather than objective performance measures or a psychometrically validated scale. Objective indicators such as rubric based proposal evaluation, budget justification, presentation performance, or panel ratings were not collected. A response shift or testing effect is possible because students may have understood the competencies differently after the activities. In addition, the first author designed and taught the activity and facilitated debriefing. Although participation was voluntary, responses were non-identifiable to the instructor, and the post assessment was completed individually on students’ own smartphones, expectancy or social desirability effects cannot be completely excluded.

Fourth, although the review process was standardized in roles, perspectives, materials, timing, questioning rules, and session flow, questions were tailored to each proposal and therefore differed in content and difficulty. The panel members had substantial administrative experience but were not current municipal public health executives, so the simulation represented relevant administrative perspectives rather than reproducing municipal public health decision making exactly.

Finally, the post assessment was conducted immediately after the program and policy development section. The findings therefore reflect short term perceived learning and do not establish retention, transfer to public health nursing practicum, or application after graduation. Future evaluations should include comparison groups, objective performance measures, and longer term follow up.

## Conclusion

The G-CHAM program and policy development section connected GIS based community health assessment with program planning, simulated executive review, and debriefing. Administrative staff with public sector experience provided students with an opportunity to encounter administrative reasoning relevant to public health nursing practice in Japan. Students reported higher self assessed competency after the section. Because this was a single group pre post evaluation based on self assessment, the findings should be interpreted as short term perceived learning rather than evidence of objective competency improvement or a causal effect of the simulated executive review. Further studies using objective performance measures, comparison groups, and longer follow up are needed.

## Data Availability

The raw data supporting the conclusions of this article will be made available by the authors, without undue reservation.
